# Effect of steady-state response versus excitatory/inhibitory balance on spiking synchronization in neural networks with log-normal synaptic weight distribution

**DOI:** 10.1007/s11571-021-09757-z

**Published:** 2021-12-03

**Authors:** Sou Nobukawa, Nobuhiko Wagatsuma, Takashi Ikeda, Chiaki Hasegawa, Mitsuru Kikuchi, Tetsuya Takahashi

**Affiliations:** 1 Department of Computer Science, Chiba Institute of Technology, 2–17–1 Tsudanuma, Narashino, Chiba 275–0016 Japan; 2 Department of Preventive Intervention for Psychiatric Disorders, National Institute of Mental Health, National Center of Neurology and Psychiatry, 4-1-1 Ogawa-Higashi, Kodaira, Tokyo 187-8551 Japan; 3 Faculty of Science, Department of Information Science, Toho University, Chiba, Japan; 4 Research Center for Child Mental Development, Kanazawa University, Kanazawa, Japan; 5 United Graduate School of Child Development, Osaka University, Kanazawa University, Hamamatsu University School of Medicine, Chiba University and University of Fukui, Osaka, Japan; 6 Department of Psychiatry and Behavioral Science, Kanazawa University, Kanazawa, Japan; 7 Department of Neuropsychiatry, University of Fukui, Yoshida, Japan; 8 Uozu Shinkei Sanatorium, Toyama, Japan

**Keywords:** Spiking neural network, Log-normal distribution, Synchronization, Excitatory-inhibitory balance, Auditory steady-state response, Steady-state visually evoked potential

## Abstract

Synchronization of neural activity, especially at the gamma band, contributes to perceptual functions. In several psychiatric disorders, deficits of perceptual functions are reflected in synchronization abnormalities. Plausible cause of this impairment is an alteration in the balance between excitation and inhibition (E/I balance); a disruption in the E/I balance leads to abnormal neural interactions reminiscent of pathological states. Moreover, the local lateral excitatory-excitatory synaptic connections in the cortex exhibit excitatory postsynaptic potentials (EPSPs) that follow a log-normal amplitude distribution. This long-tailed distribution is considered an important factor for the emergence of spatiotemporal neural activity. In this context, we hypothesized that manipulating the EPSP distribution under abnormal E/I balance conditions would provide insights into psychiatric disorders characterized by deficits in perceptual functions, potentially revealing the mechanisms underlying pathological neural behaviors. In this study, we evaluated the synchronization of neural activity with external periodic stimuli in spiking neural networks in cases of both E/I balance and imbalance with or without a long-tailed EPSP amplitude distribution. The results showed that external stimuli of a high frequency lead to a decrease in the degree of synchronization with an increasing ratio of excitatory to inhibitory neurons in the presence, but not in the absence, of high-amplitude EPSPs. This monotonic reduction can be interpreted as an autonomous, strong-EPSP-dependent spiking activity selectively interfering with the responses to external stimuli. This observation is consistent with pathological findings. Thus, our modeling approach has potential to improve the understanding of the steady-state response in both healthy and pathological states.

Introduction

Synchronization of neural activity with external periodic stimuli in the sensory cortex is observed with phenomena such as the auditory steady-state response and the steady-state visually evoked potential captured by techniques such as electroencephalography and magnetoencephalography (Galambos et al. 1981; Naatanen and Näätänen 1992; Hillyard et al. 1995). This synchronization reaches significant levels in the gamma frequency band, which plays a role in perception (Galambos et al. 1981; Naatanen and Näätänen 1992; Hillyard et al. 1995). Conversely, deficits in perception and transfer of perceptual information within and between cortical areas are reflected in abnormalities in the steady-state response (O’Donnell et al. 2004; Wilson et al. 2007; Spencer et al. 2008; Rass et al. 2010; Rojas et al. 2011; Oda et al. 2012; McNally and McCarley 2016; Zhou et al. 2018). Clinically, the steady-state response, which is defined as a neural response against a steady periodic stimulus, especially the auditory steady-state response, is widely used for assessing psychiatric disorders such as schizophrenia (O’Donnell et al. 2013; McNally and McCarley 2016; Zhou et al. 2018), bipolar disorder (O’Donnell et al. 2004; Spencer et al. 2008; Rass et al. 2010; Oda et al. 2012), and autism spectrum disorder (Wilson et al. 2007; Rojas et al. 2011; Seymour et al. 2020). To quantify the steady-state response, previous studies (Tan et al. 2015; Legget et al. 2017; Seymour et al. 2020) used the inter-trial phase coherence (ITPC), i.e., the degree of phase synchronization among trials in which the same stimulus was applied (Tallon-Baudry et al. 1996).

Physiological and modeling studies have shown that gamma-band oscillations are produced by the interaction between excitatory and inhibitory neuron populations in local neuronal circuits (Penttonen et al. 1998; Izhikevich 2003; Hájos and Paulsen 2009). More specifically, activation of the excitatory neuron population through excitatory-excitatory synaptic connections is suppressed by the strong and rapid feedback from inhibitory neurons. Activation and suppression are repeated to produce the gamma oscillation, known as the pyramidal-interneuron gamma (PING) (Börgers and Kopell 2003). Whereas in response to a tonic excitation stimulus, the fast inhibitory synaptic connections in the inter inhibitory neural networks also produce gamma-band oscillations, known as interneuronal gamma (ING) (Whittington et al. 2000; Bartos et al. 2002). In several psychiatric disorders, an impairment in the function of inhibitory synaptic connections has been reported in relation to deficits in gamma activity (Ben-Ari 2015; McNally and McCarley 2016) caused by abnormal spatiotemporal interactions of neural activity (Uhlhaas et al. 2008; Hirano et al. 2015; Uhlhaas and Singer 2012), such as reduction in gamma-functional connectivity in schizophrenia (Takahashi et al. 2018), enhancement of gamma-functional connectivity in autism spectrum disorder (Takahashi et al. 2017), and significant reduction in the steady-state response of gamma-activity in schizophrenia (McNally and McCarley 2016; Zhou et al. 2018) and autism spectrum disorder (Wilson et al. 2007; Rojas et al. 2011; Seymour et al. 2020). Although the causes of these abnormalities remain unclear, an alteration in the balance between excitation and inhibition (E/I balance) is a major contributing factor(Gibson et al. 2008; Chao et al. 2010; Chattopadhyaya and Di Cristo 2012; Glausier and Lewis 2013). Powell et al. found that deficits in either the production or migration of GABAergic inhibitory neurons decrease the number of GABAergic inhibitory neurons in the cortex. Consequently, the cortical neural network becomes hyper-excitable (Powell et al. 2003). Hashemi et al. revealed a smaller inhibitory neural population in autism spectrum disorder with impairment of gamma-band oscillation compared to that of typical development (Hashemi et al. 2017). Rubin et al. showed that an external perturbation preventing efficient learning and memorization was canceled in an optimized, E/I-balanced network, suggesting that the function of the E/I balance is to neutralize such perturbations (Rubin et al. 2017). Dehghani et al. demonstrated that a break in the E/I balance leads to an abnormal temporal interaction between excitatory and inhibitory neuron populations reminiscent of pathological states (Dehghani et al. 2016). Therefore, an E/I imbalance impairs the production of gamma-band oscillations such as PING and ING; the resultant abnormal gamma-band oscillations could lead to pathological conditions and brain dysfunction (Gibson et al. 2008; Chao et al. 2010; Chattopadhyaya and Di Cristo 2012; Glausier and Lewis 2013). In modeling studies regarding E/I balance and the spatiotemporal patterns of neural activity, Guo et al. highlighted that inhibitory synaptic factors, such as inhibitory synaptic weights and their synaptic delay play a crucial role in controlling spatiotemporal neural activity (Guo et al. 2012, 2016a, b). Therefore, a detailed evaluation of the influence of the E/I balance on gamma-band oscillations through modeling analysis is important to understand the mechanisms behind the alternation of gamma-band oscillations observed in pathological conditions and brain dysfunction.

In the synaptic networks of the cerebral cortex, excitatory pre-synaptic neurons’ spikes increase the membrane potential of postsynaptic neurons, which are called excitatory postsynaptic potentials (EPSPs). Most synapses in the local and lateral excitatory-excitatory synaptic connections of the cortex produce EPSPs of sub-millivolt amplitude, while a minority produce large EPSPs ($\gtrsim 1.0$ mV) (Song et al. 2005; Lefort et al. 2009). The amplitudes of these EPSPs follow a long-tailed distribution, specifically, a log-normal distribution. Teramae et al. focused on this long-tailed distribution (Teramae et al. 2012) for modeling spontaneous cortical activity, typically characterized by irregular neuron spiking and a low firing rate ($\approx 1$ Hz) but a highly synchronous spike transmission between specific neurons (Softky and Koch 1993; Hromádka et al. 2008; Sakata and Harris 2009). Particularly, spikes’ transmission in response to spikes propagating to the minority of strong synapses is enhanced by spikes propagating to the majority of weak synapses as noise. Thus, spontaneous activity is said to be produced by stochastic resonance. This model of spontaneous activity has been widely used for clarifying the function of cortical neural networks and further expanded upon (Hiratani et al. 2013; Omura et al. 2015; Kada et al. 2018; Nobukawa et al. 2019, 2020). For example, a log-normal distribution of EPSPs may enhance learning and memory (Hiratani et al. 2013; Omura et al. 2015). Furthermore, by incorporating the dual nature of complex network structures observed in the cortex (Watanabe et al. 2016) into a spiking neural network with a long-tailed EPSP distribution, the spatiotemporally complex neural activity of the real cortex was recently reproduced (Kada et al. 2018; Nobukawa et al. 2019). Hence, a long-tailed distribution of EPSPs in local cortical networks is an important factor for determining the spatiotemporal characteristics of neural activity and enhancing brain functionality. However, only a few studies have assessed the role of the log-normal EPSP distribution in pathological neuronal behavior.

Accordingly, we hypothesized that a long-tailed EPSP distribution under an abnormal E/I balance might generate a pathological neural behavior and subsequent deficits in perception and inaccuracy of synaptic transmission in individuals with psychiatric disorders. To validate this hypothesis, we aimed to evaluate the synchronization of neural activity with an external periodic stimulus in a spiking neural network model in cases of both E/I balance or imbalance with or without a long-tailed EPSP distribution. Specifically, we evaluated the synchronization to high and low frequencies by performing analyses of the power spectrum and ITPC in cases with E/I ratios of 3/1 to 9/1.

## Material and methods

### Spiking neural network

In this study, we used a spiking neural network with synaptic weights following a log-normal distribution, as modeled by Teramae et al. (2012). The membrane potential *v*(*t*) in the network is described using a leaky-integrate-and-fire neuron model:

1 $$\begin{aligned} \frac{dv}{dt}= & {} -\frac{1}{\tau _m}(v-V_L)-g_E(v-V_E)-g_I(v-V_I)+I_{\text {ex}}, \end{aligned}$$

2 $$\begin{aligned} {\text {if }}v\ge & {} V_{{\text {thr}}}\, {\rm mv},\,{\rm then}\, v(t) \rightarrow V_r. \end{aligned}$$

**Fig. 1:**
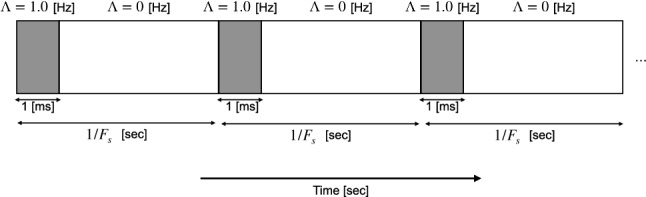
Input rate $\varLambda$ Hz for Poisson process to produce an external input signal $I_{\text {ex}}$ with a frequency $F_s$ Hz. Under this input signal, the firing rate directly evoked by $I_{\text {ex}}$ in each neuron approximates to $F_s \cdot \varLambda /10^3$ Hz

Here $\tau _m$ is the membrane decay constant; $V_E$, $V_I$, and $V_L$ are the reversal potentials of the AMPA-receptor-mediated excitatory synaptic current, the inhibitory synaptic current, and the leak current, respectively; $V_r$ is the reset voltage, and $V_{\text {thr}}$ is the threshold voltage. In this study, the periodic external input signal $I_{\text {ex}}$ with a frequency $F_s$ Hz is applied. At the beginning of the input period $1/F_s$ sec with a time-window of 1.0 ms, $I_{\text {ex}}$ is $21\cdot \delta (t-t_{\text {ex}})$ mV, where the input times $t_{\text {ex}}$ are drawn from Poisson distribution with an input rate $\varLambda =1.0$ Hz. Under the strength of dirac delta function $\delta$: 21 and condition for $V_{\text {thr}}-V_L<21$, the single spike of external input evoke the spikes in Eq. (1). During the rest of the input period, $\varLambda$ becomes 0 Hz, consequently, $I_{\text {ex}}=0$. This method to produce an external input signal $I_{\text {ex}}$ is shown in Fig. 1. Hence, the firing rate directly evoked by $I_{\text {ex}}$ in each neuron approximates to $F_s \cdot \varLambda /10^3$ Hz. The dynamic behaviors of the excitatory $g_E(t)$ $\hbox {ms}^{-1}$ and inhibitory synaptic conductances $g_I(t)$ $\hbox {ms}^{-1}$ are described as follows:

3 $$\begin{aligned} \frac{dg_X}{dt}=-\frac{g_X}{\tau _s}+\sum _jG_{X,j}\sum _{s_j}\delta (t-s_j-d_j),~~~X=E,I. \end{aligned}$$

Here $\tau _s$ is the decay constant of the excitatory and inhibitory synaptic conductances; $s_j$, $d_j$, $G_{E,j}$, and $G_{I,j}$ are the spike times of the synaptic inputs from the *j*-th neuron, the synaptic delays, and the synaptic weights of excitatory and inhibitory synapses, respectively. Additionally, the excitatory synaptic weight $G_{E,j}$ is classified to the excitatory-to-excitatory synaptic weight $G_{EE,j}$ and the excitatory-to-inhibitory synaptic weight $G_{EI,j}$; the inhibitory synaptic weight $G_{I,j}$ is classified to the inhibitory-to-excitatory synaptic weight $G_{IE,j}$ and the inhibitory-to-inhibitory synaptic weight $G_{II,j}$. The parameter set was as follows: $V_L=-70$ mV, $V_E=0$ mV, $V_I=-80$ mV, $V_r=-60$ mV, $V_{\text {thr}}=-50$ mV, $\tau _m=20$ ms (excitatory neuron), $\tau _m=10$ ms (inhibitory neuron), and $\tau _s=2$ ms (Teramae et al. 2012). To solve Eqs. (1)–(3), we used the Euler method with time step $\varDelta t=0.1$ ms. The refractory period was set to 1 ms. Synaptic delays were set to uniform random values of [1, 3] ms at the excitatory-to-excitatory connections and [0, 2] ms at all the other connections. The excitatory and inhibitory neurons had a total network size of 12000, and the respective sizes of their neural populations (indicated by $N_E$ and $N_I$, respectively) were determined by the E/I ratios. Each neuron was randomly connected with a coupling probability fixed by excitatory and inhibitory neurons. This coupling probability is defined by the probability of the objective neuron coupling with postsynaptic neurons. In this study, the probability for excitatory neurons was 0.1, and that for inhibitory neurons was 0.5. The network was reconstructed using different random seeds across trials.

**Fig. 2:**
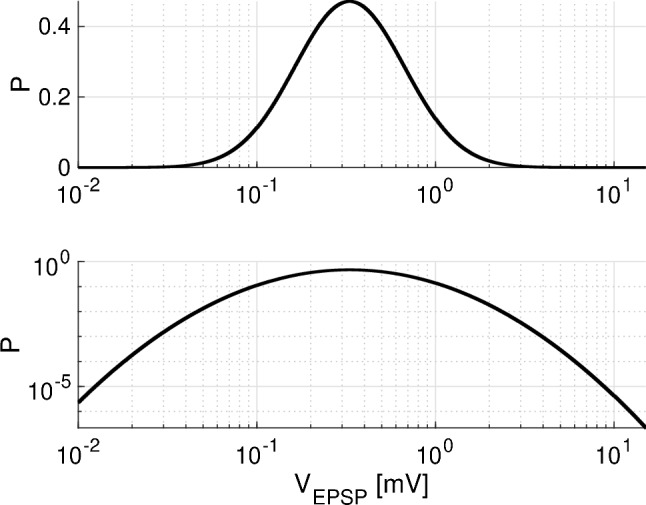
Distribution of probability density for excitatory post-synaptic potentials (EPSPs) $V_{\text {EPSP}}$ in excitatory-excitatory connections. The upper and lower panels show semi-logarithmic and double logarithmic charts, respectively. The parameters are set to $\sigma =1.0$, and the distribution mode is set to $\mu -\sigma ^2=\log 0.2$. In this study, we compared the spiking neural network activity between excitatory-to-excitatory connections with strong EPSPs $V_{\text {EPSP}}>9$ mV and without. For each excitatory neuron, the number of synapses with $V_{\text {EPSP}}>9$ mV is approximately one

The EPSP amplitude $V_{\text {EPSP}}$ mV, which is an increase in the membrane potential from the resting state due to the excitatory synaptic input, was derived from a log-normal distribution. This probability density is given by

4 $$\begin{aligned} p(x)=\frac{\exp [-(\log x-\mu )^2/2\sigma ^2]}{\sqrt{2\pi }\sigma x}, \end{aligned}$$

where *x* indicates the $V_{\text {EPSP}}$ mV as the random variable. We set $\sigma =1.0$ and the mode of the distribution $\mu -\sigma ^2=\log 0.2$. This parameter set, *p*(*x*), is shown in Fig. 2. Unrealistically large values for $V_{\text {EPSP}}$ exceeding 20 mV were rejected, and new values were drawn from the distribution. In studies reporting physiological experiments, the amplitude of synaptic coupling with log-normal distribution was measured by EPSPs (Song et al. 2005; Lefort et al. 2009). Therefore, this observable value $V_{\text {EPSP}}$ must be translated into a synaptic weight $G_E$. For this purpose, we consider the case where a post-synaptic neuron receives a spike input from a single excitatory synapse at $t=0$ ms. The dynamics of the membrane potential *v*(*t*) can then be described as

5 $$\begin{aligned} \frac{dv(t)}{dt}= & {} -\frac{1}{\tau _m}(v(t)-V_L)-g_E(t)(v(t)-V_E), \end{aligned}$$

6 $$\begin{aligned} \frac{dg_E(t)}{dt}= & {} -\frac{g_E(t)}{\tau _s}+G_E\delta (t). \end{aligned}$$

In solving Eqs. (5) and (6) numerically, the relationship between the rising voltage of *v*(*t*) from $v=V_L(=-70)$ mV and the amplitude $G_E$ is derived. Consequently, $G_{EE}$ can be set to $V_{\text {EPSP}}/100$, where $V_{\text {EPSP}}$ is obtained from the stochastic process following Eq. (4). The excitatory synaptic weight in excitatory-to-inhibitory neurons $G_{EI}$, the inhibitory synaptic weight in the inhibitory-to-excitatory $G_{IE}$, and the inhibitory synaptic weight in inhibitory-to-inhibitory neurons $G_{II}$ were set to the constant values of $G_{EI}=0.018$ and $G_{IE,II}=0.002,0.0025$, respectively (Teramae et al. 2012). In synaptic connections among excitatory-to-excitatory neurons, spike transmission fails at a rate depending on EPSP amplitude as follows: $P_E=\frac{a}{a+V_{\text {EPSP}}}$  ($a=0.1$ mV) (Teramae et al. 2012). In our previous studies (Nobukawa et al. 2019, 2020) and that by Teramae et al. (2012), the spontaneous activity under the condition with E/I ratio of 4/1 was evaluated. In this study, we addressed the steady-state response against periodic stimuli under several E/I ratios.

For comparison, we calculated the spiking neural network activity without excitatory-to-excitatory connections capable of producing strong EPSPs, i.e., $V_{\text {EPSP}}>9$ mV. For each excitatory neuron, the number of synapses with $V_{\text {EPSP}}>9$ mV is approximately one. Therefore, this setting corresponds to the removal of the maximum EPSP synapse in each neuron. The program code for spiking the neural network used in this study was developed in Brian2 (https://brian2.readthedocs.io/en/2.0rc/index.html) (Goodman et al. 2014). The source code for simulating the spiking activity can be found at the following address: https://github.com/SouNobukawa/ASSR_SNN.

### Evaluation indices

#### Method of observing neural activity

To observe a time-dependent spiking activity, we defined the spiking-rate time series for the excitatory ($r_E$ Hz) and inhibitory ($r_I$ Hz) neuron populations as follows:

7 $$\begin{aligned} r_X(t)=1000~\frac{S_X(t)}{\varDelta t~N_X},~~~X=E,I. \end{aligned}$$

$S_E$ and $S_I$ indicate the number of spikes in the excitatory and inhibitory neural populations, respectively, at each time step within $\varDelta t=0.1$ ms. In this study, $r_E(t)$ and $r_I(t)$ were smoothed using a Gaussian-shaped window with $\sigma =10$ ms. This $\sigma$ value was determined by confirming the timescale of the membrane decay constant.

#### Power spectrum analysis

To analyze the power spectrum of the spiking activity, the z-scored time series $r_{E}$ during the interval [3, 7] sec was analyzed. By this process, the constant component and the effect of the range of variation of $r_{E}$ were removed from the power spectrum profile. The power spectrum analysis was based on 10 trials. We used different seeds for random values to construct networks, initial membrane potential values, and synaptic conductance across trials.

#### Inter-trial phase coherence analysis

The ITPC has been widely used to observe the steady neural response against periodic stimulus (steady-state response) as a degree of phase synchronization among trials (Tan et al. 2015; Legget et al. 2017; Seymour et al. 2020). The ITPC is calculated as follows (Tallon-Baudry et al. 1996):

8 $$\begin{aligned} {\text {ITPC}}(T,f)=\left| \frac{1}{T}\sum ^{T}_{m=1}\frac{F_m(f)}{|F_m(f)|}\right| . \end{aligned}$$

Here $F_m(f)$ and *T* represent the phase output of the Fourier transform of $r_E$ at the *m*-th trial as a function of frequency *f* Hz and evaluation duration. ${\text {ITPC}}$ approaches 1.0, which corresponds to complete phase synchronization. Since the input signal is fixed across trials, ${\text {ITPC}}\approx 1.0$ means that the phase of $r_E$ synchronizes exactly to the input signal.

## Results

**Fig. 3:**
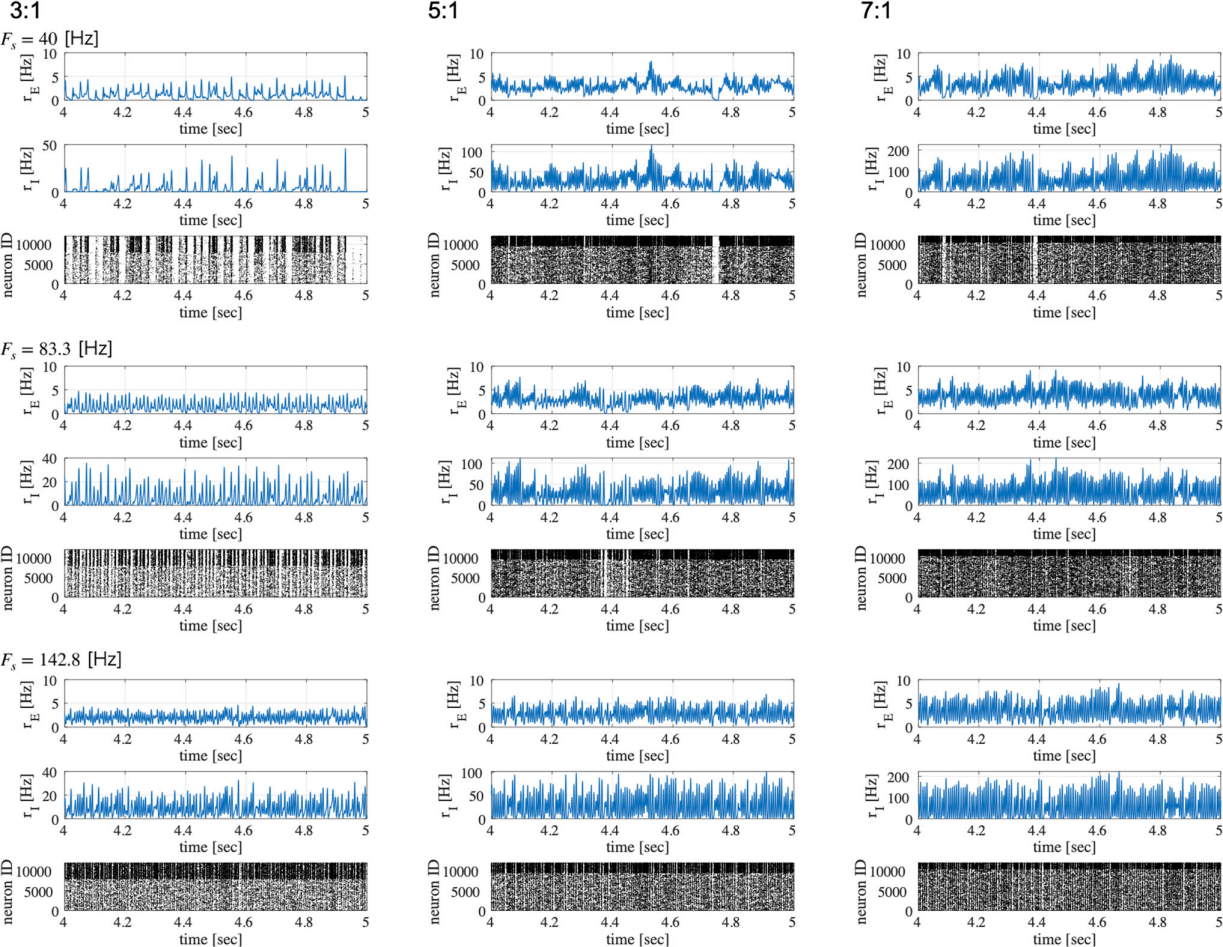
Modeled spiking activity during periodic synaptic input under different ratios of the excitatory to inhibitory neurons ($N_E:N_I$)(3 : 1, 5 : 1, and 7 : 1) in the presence of strong synaptic excitatory-to-excitatory neuron connections $V_{\text {EPSP}}>9$ mV capable of generating large-amplitude excitatory post-synaptic potentials (EPSPs). The upper three rows show, respectively, the spiking-rate time series of the excitatory and inhibitory neuron populations, and the raster plot of the neuron populations (excitatory below), all with input frequency $F_s=40$ Hz. The middle three rows show the results for $F_s=83.3$ Hz, and the lower three rows show the results for $F_s=142.8$ Hz. With an increasing E/I ratio, the spiking activity represented by the spiking rate time-series of $r_E$ and $r_I$ and raster plot becomes irregular. ($V_L=-70$ mV, $V_E=0$ mV, $V_I=-80$ mV, $V_r=-60$ mV, $V_{\text {thr}}=-50$ mV, $\tau _m=20$ ms (excitatory neuron), $\tau _m=10$ ms (inhibitory neuron), $\tau _s=2$ ms, $G_{IE}=0.002,G_{II}=0.0025,G_{EI}=0.018$)

**Fig. 4:**
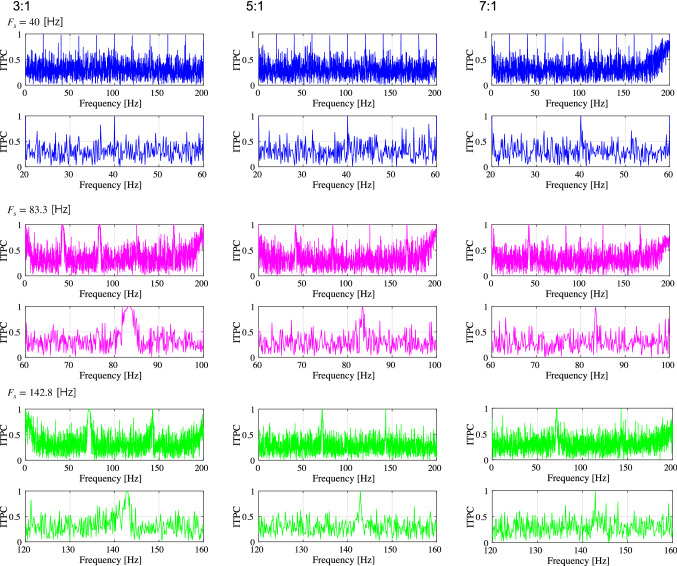
Inter-trial phase coherences (ITPC) of the spiking-rate time series of excitatory neuron populations corresponding to the results shown in Fig. 3. The upper two rows show, respectively, the profile in the range [0, 200] Hz and the magnified profile in the range around $F_s$ for $F_s=40$ Hz. The middle two rows show the results for $F_s=83.3$ Hz, and the lower two rows show the results for $F_s=142.8$ Hz. A peak is observed in the ITPC around the input stimulus frequency at all E/I ratios as the response to the input stimulus

**Fig. 5:**
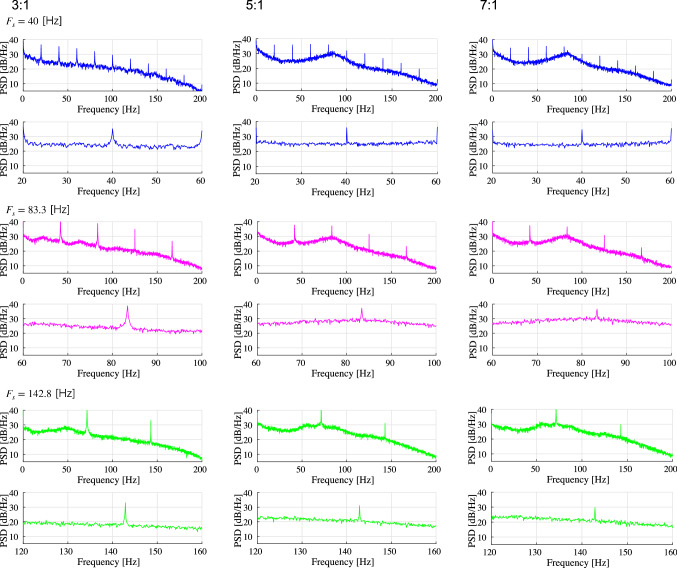
Power spectra density (PSD) of the spiking-rate time series of the excitatory neuron population corresponding to the results shown in Fig. 3. The upper two rows show, respectively, the profile in the range [0, 200] Hz and the magnified profile in the range around $F_s$ for $F_s=40$ Hz. The middle two rows show the results for $F_s=83.3$ Hz, and the lower two rows show the results for $F_s=142.8$ Hz. A peak is observed in the power spectrum around the input stimulus frequency at all E/I ratios as the response to the input stimulus

**Fig. 6:**
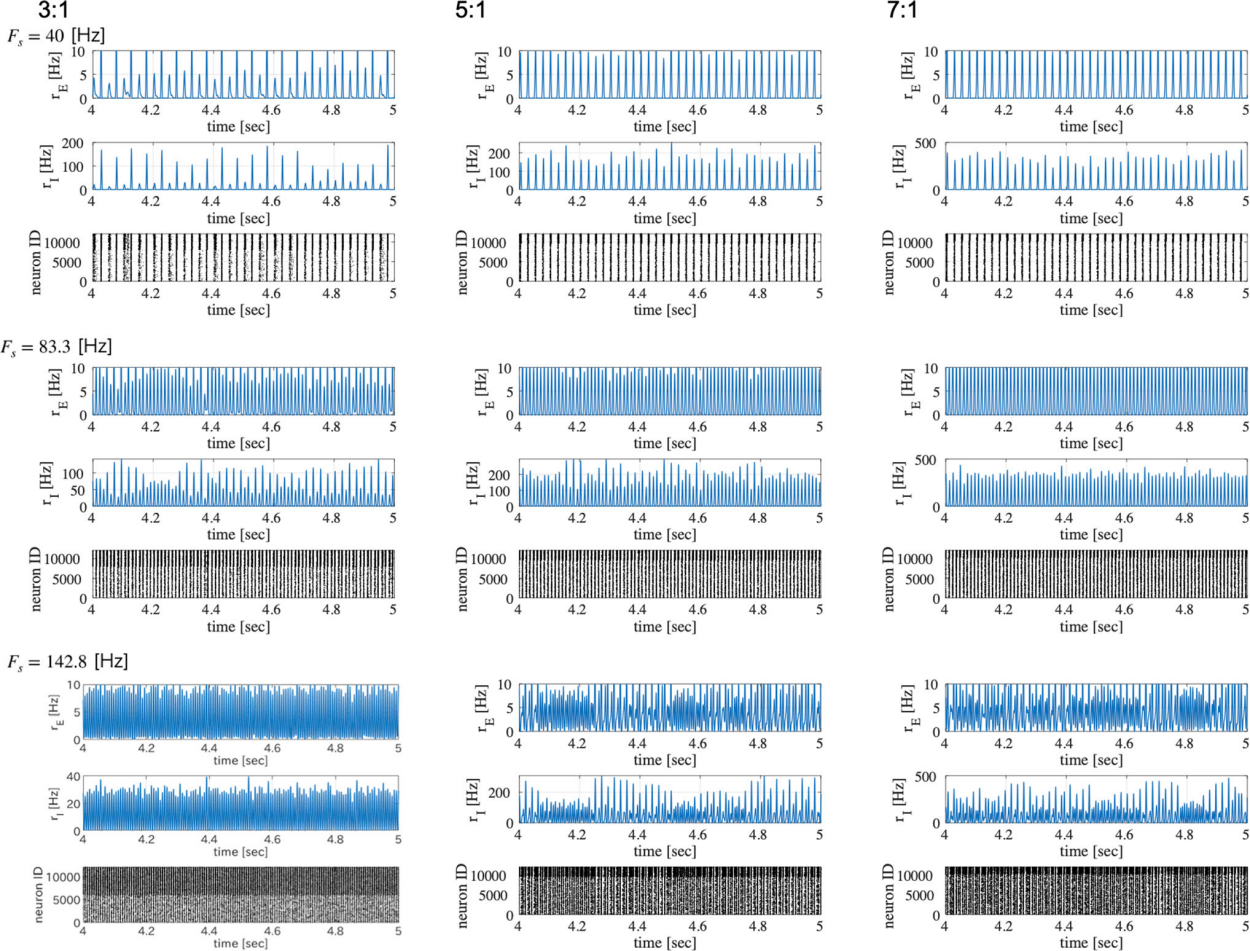
Modeled spiking activity during periodic synaptic input under different ratios of excitatory to inhibitory neurons ($N_E:N_I$; 3 : 1, 5 : 1, and 7 : 1) without strong synaptic excitatory-to-excitatory neuron connections (i.e., without large EPSPs $V_{\text {EPSP}}>9$ mV). The upper three rows show the spiking-rate time series of the excitatory and inhibitory neuron populations and the raster plot of the neuron populations (excitatory below) at the input frequency of $F_s=40$ Hz. The middle three rows show the corresponding results for $F_s=83.3$ Hz, and the lower three rows show the corresponding results for $F_s=142.8$ Hz. The regularity of spiking activity is relatively maintained with an increasing E/I ratio in comparison to the case with strong EPSPs (see Fig.3). ($V_L=-70$ mV, $V_E=0$ mV, $V_I=-80$ mV, $V_r=-60$ mV, $V_{\text {thr}}=-50$ mV, $\tau _m=20$ ms (excitatory neuron), $\tau _m=10$ ms (inhibitory neuron), $\tau _s=2$ ms, $G_{IE}=0.002,G_{II}=0.0025,G_{EI}=0.018$)

**Fig. 7:**
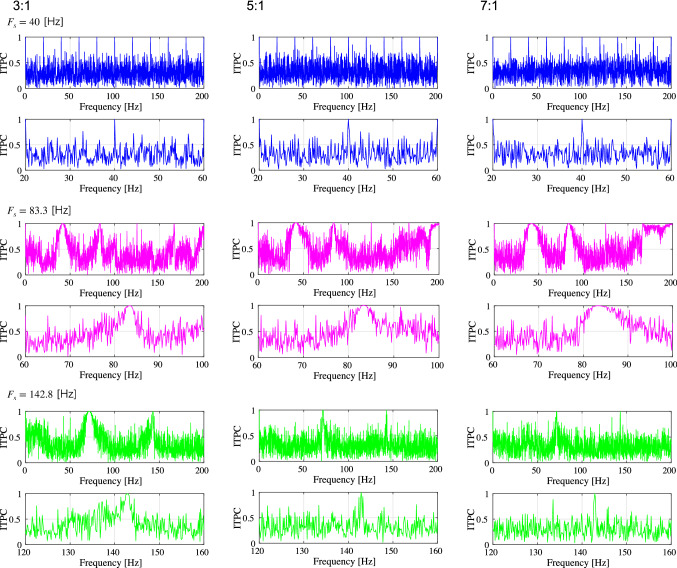
Inter-trial phase coherences (ITPC) of the spiking-rate time series of the excitatory neuron population corresponding to the results shown in Fig. 6. The upper two rows show, respectively, the profile in the range [0, 200] Hz and the magnified profile in the range around $F_s$ for $F_s=40$ Hz. The middle two rows show the results for $F_s=83.3$ Hz, and the lower two rows show the results for $F_s=142.8$ Hz. A peak in the ITPC at around the input stimulus frequency is also observed

**Fig. 8:**
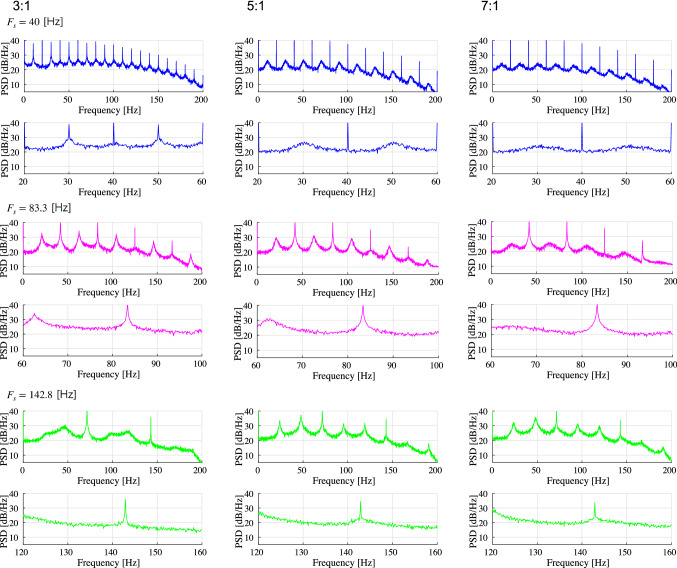
Power spectra density (PSD) of the spiking-rate time series of the excitatory neuron population corresponding to the results shown in Fig. 6. The upper two rows show respectively the profile in the range [0, 200] Hz and the magnified profile in the range around $F_s$ for $F_s=40$ Hz. The middle two rows show the results for $F_s=83.3$ Hz, and the lower two rows show the results for $F_s=142.8$ Hz. A peak in the power spectrum at around the input stimulus frequency is observed as well

First, we evaluated the spiking activity during the periodic synaptic input under different conditions of the excitatory-inhibitory balance. Figure 3 shows the spiking-rate time series of excitatory and inhibitory neuron populations as well as raster plots for input frequencies $F_s=40$, 83.3, and 142.8 Hz and ratios of excitatory to inhibitory neurons ($N_E:N_I$) = 3 : 1, 5 : 1, and 7 : 1. In our simulation, the input stimulus is controlled by a 1 ms time-window; therefore, the input frequencies $F_s=40$, 83.3, and 142.8 Hz correspond to periods of 25, 12, and 7 ms, respectively. With an increasing E/I ratio, the spiking activity represented by the spiking rate time-series of $r_E$ and $r_I$ and raster plot becomes irregular. The ITPC and power spectrum of the excitatory neuron population are shown in Figs. 4 and 5, respectively. In these results, a peak is observed in the ITPC and power spectrum around the input stimulus frequency at all E/I ratios as the response of the input stimulus. Figure 6 represents the spiking-rate time series in the control case without strong EPSPs. The regularity of spiking activity is relatively maintained with an increasing E/I ratio in comparison to the case with strong EPSPs. Figures 7 and 8 show the corresponding ITPC and power spectrum. Consequently, a peak in the ITPC and power spectrum at around the input stimulus frequency is also observed. In the overview of time-series and power spectra and ITPC profiles, the significance of the difference between cases with and without strong EPSPs could not be confirmed.

**Fig. 9:**
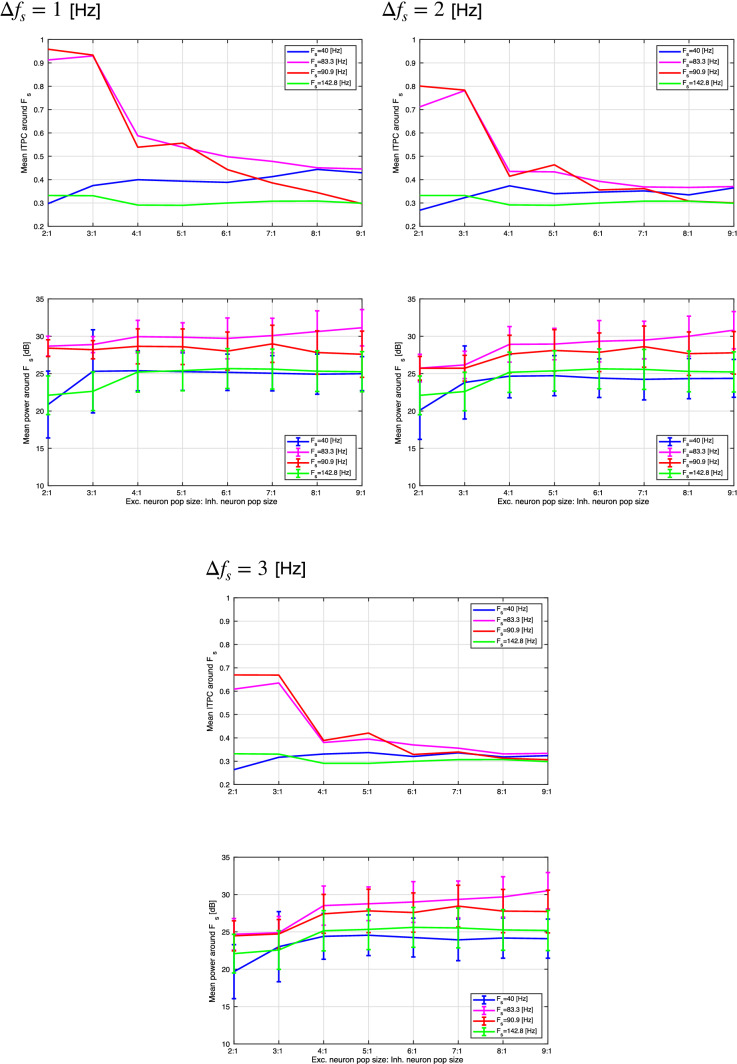
(Upper) Mean ITPC in the excitatory neuron population around the input frequency $F_s$ at four input frequencies ($F_s=40,83.3,90.9$, and 142.8 Hz) in the case of strong synaptic connections. (Lower) Mean power spectrum of the peristimulus time histogram around the input frequency $F_s$ at the same frequencies. $\varDelta F_s$ is set to 1.0, 2.0, and 3.0 Hz, respectively. At $F_s=40,142.8$ Hz, the ITPC and mean power maintain constant values with an increasing E/I ratio. However, at $F_s=83.3,90.9$ Hz, the ITPC markedly decreases with an increasing E/I ratio. Bars indicate the standard deviation across ten trials

**Fig. 10:**
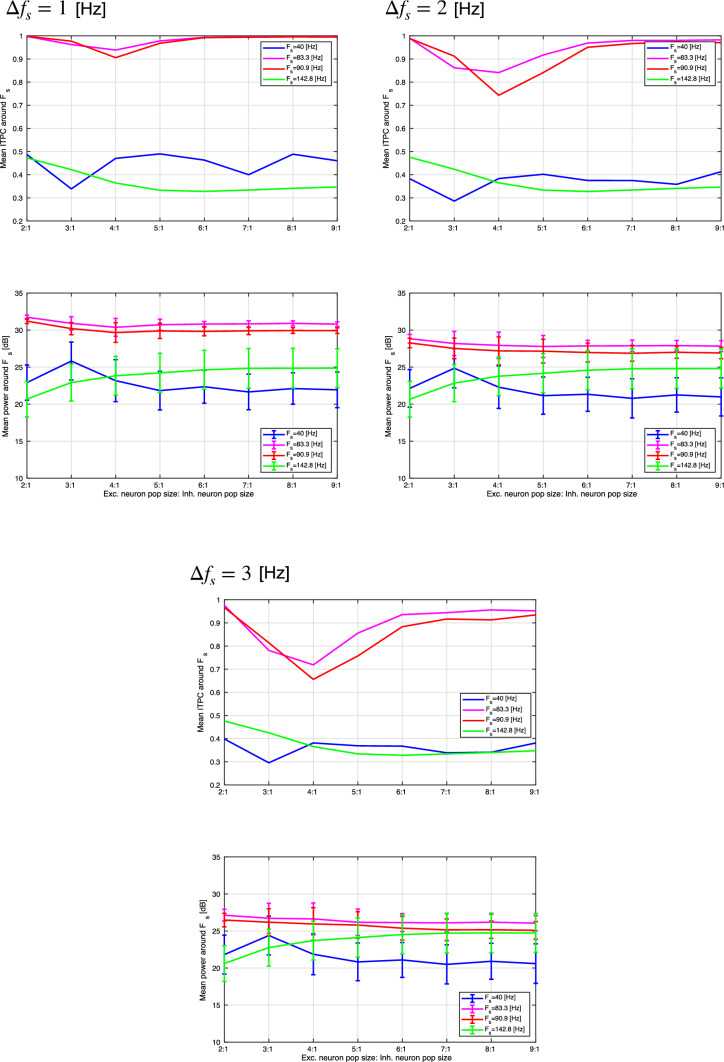
(Upper) Mean ITPC in the excitatory neuron population around the input frequency $F_s$ at four input frequencies ($F_s=40,83.3,90.9$, and 142.8 Hz) in the case without strong synaptic connections. (Lower) Mean power spectrum of the peristimulus time histogram around the input frequency $F_s$ at the same frequencies. $\varDelta F_s$ is set to 1.0, 2.0, and 3.0 Hz, respectively. At $F_s=40,142.8$ Hz, the ITPC and mean power maintain constant values with an increasing E/I ratio. However, at $F_s=83.3,90.9$ Hz, the ITPC minimizes at the E/I ratio $(N_E:N_I)$ = 4 : 1. The monotonic decrease with an increasing E/I ratio, which arises in the case with strong synaptic connections shown in Fig. 9, is not observed in the case without strong synapses. Bars indicate the standard deviation across ten trials

To evaluate the profiles of power spectra and ITPC in more detail, Fig. 9 shows the mean ITPC of the excitatory neuron population around the input frequency $F_s$ in the range $[F_s-\varDelta f_s,F_s+\varDelta f_s]$ Hz ($\varDelta f_s=1,2,3$ Hz). (The same frequency interval is used to compute all ITPCs and power spectra.) These results show that the ITPC and mean power at $F_s=40,142.8$ Hz maintain a constant value with an increasing E/I ratio. At $F_s=83.3,90.9$ Hz, the ITPC decreases markedly with an increasing E/I ratio. This tendency is maintained in all $\varDelta f_s$ cases. In the absence of strong synaptic connections at $F_s=40,142.8$ Hz (see Fig. 10), the ITPC and mean power maintain constant values with an increasing E/I ratio. At $F_s = 83.3,90.9$ Hz, the ITPC minimizes at the E/I ratio $(N_E:N_I)$ = 4 : 1. Hence, the monotonic decrease with an increasing E/I ratio, which arises in strong synaptic connections at $F_s=83.3,90.9$ Hz, is not observed in the absence of strong synapses.

## Discussion and conclusion

In this study, we evaluated the synchronization of spiking activity with periodic stimuli in two types of spiking neural networks using different network E/I ratios. The first one includes synapses capable of producing strong (i.e., large-amplitude) EPSPs as a log-normal EPSPs distribution, and the second one is a network without strong EPSPs. The power spectrum and ITPC results show that when using external periodic stimuli of a relatively high frequency, the degree of synchronization decreases with an increasing E/I ratio in the presence of strong EPSPs. However, in the absence of strong EPSPs, this monotonic reduction of the ITPC does not emerge.

We must first consider why the monotonic reduction of ITPC arises in the presence of strong EPSPs. Under conditions of low E/I ratio or absence of strong EPSPs, the number of synapses with strong EPSPs is low. In such networks, spikes induced by external stimuli arise, but autonomous spiking due to mutual driving of neurons rarely arises. However, under the condition of a high ratio of excitatory to inhibitory neuron numbers and the presence of strong EPSPs, autonomous spiking triggered by the external stimulus can persist and be maintained even with the removal of the external stimulus (Teramae et al. 2012). These autonomous spikes disturb the phase coherence among trials, causing a decrease in the ITPC with an increasing E/I ratio visible only in the presence of strong-EPSPs.

Furthermore, we must consider why the synchronization can become dependent on the frequency of the input stimulus. In our previous study, autonomous spiking activity in a network with strong EPSPs exhibited dynamic neural oscillations with high-frequency components ($\approx 80$ Hz) under conditions of random network topology (corresponding to the network topology used in the present study) (Nobukawa et al. 2019). Moreover, in our spiking neural network with strong-EPSP, the high-frequency component of autonomous neural activity involving the gamma band activity under an absent external stimulus is enhanced with increasing E/I ratio (see “Appendix” section). Therefore, the monotonic reduction in synchronization observed with an increasing E/I ratio can be interpreted as an autonomous, strong-EPSP-dependent spiking activity selectively interfering with the responses to external stimuli, which have a high-frequency component.

This finding is supported by studies of resting-state neural activity and steady-state responses under pathological conditions (Grent et al. 2018). In patients with schizophrenia, a recent study of resting-state neural activity demonstrated that activity in the low- and high-gamma bands ($60-90$ Hz) is enhanced by increasing the excitatory ratio (Grent et al. 2018). This enhanced autonomous neural activity at gamma frequencies leads to a reduction in the synchronization with high-frequency external stimuli (McNally and McCarley 2016; Zhou et al. 2018). In patients with autism spectrum disorder, the enhanced excitatory neuron activity induced by dysfunction in local inhibitory neurons leads to abnormal autonomous gamma-band neural activity (Spence and Schneider 2009; Berg and Plioplys 2012) (reviewed in Kessler et al. (2016)). As in cases of schizophrenia, this abnormal gamma hyperactivity leads to a reduction in synchronization with high-frequency stimuli (Wilson et al. 2007; Rojas et al. 2011; Seymour et al. 2020). These pathological findings are congruent with our simulation results (see Fig. 9). Therefore, we consider a spiking neural network connected by synapses following a log-normal EPSP distribution to be a potentially influential model for describing the steady-state response under pathological conditions.

A few limitations of this study must be considered. The spiking neural network used in this study does not model functional inter-regional connections. However, incorporating region-specific sub-networks and inter-regional connections into this modeling approach might be useful for revealing the steady-state response in greater detail. Additionally, the effect of voltage-dependent currents was not considered in the modeling of EPSPs of this study. This characteristic might affect the steady-state response. Further, the parameters for neuron and synapse were simplified in this study; e.g., common settings were used for the decay constants of excitatory and inhibitory synaptic conductances. However, these parameters should be adjusted to correspond to more physiological values and resemble actual cortical neural networks. Regarding how the E/I balance changed, we modified the sizes of the excitatory and inhibitory neural populations. However, there is another method for changing the amplitude of inhibitory synaptic conductance (Gao et al. 2017). The change in the dependency of steady-state responses on E/I balance according to that method should be evaluated and compared with the results obtained in this study. Moreover, in this study, the input stimulus is a relatively periodical input spike-series produced by a Poisson process. However, in real neural networks, through the neural processing pathway of auditory and visual stimuli, the input stimuli to the cortical neural network might form spiking signals of greater complexity than the periodic spikes used in this study (Gray et al. 1989; Juergens et al. 1999; Bruns and Eckhorn 2004; Nobukawa et al. 2020). Therefore, evaluating the steady-state response against more complex external stimuli may validate the findings of this study and reveal new insights into the steady-state response.

In conclusion, we evaluated the steady-state synchronization of neural activity with external periodic stimuli by an E/I-balanced, spiking neural network that followed a log-normal weight distribution at excitatory synapses. We found that strong synaptic connections produced a monotonic reduction in synchronization with an increase in the ratio of excitatory to inhibitory neuron populations for a high-frequency input stimulus. This tendency might be caused by the enhancement of autonomous gamma-band activity by synapses with strong EPSPs. This result is congruent with pathological findings in schizophrenia and autism spectrum disorder. A future combination of this modeling approach with neuroimaging measures of neural network activity and whole-brain network modeling would improve the understanding of the steady-state response under healthy and pathological conditions.

## Figures and Tables

**Fig. 11:**
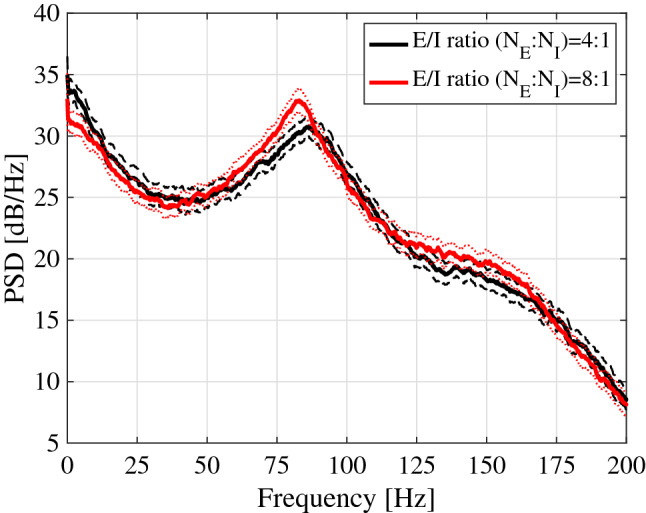
PSD of the spiking-rate time series of the excitatory neuron population in cases with an E/I ratio of 4/1 and 8/1 under absence of external input condition. Bars indicate the standard error across 10 trials. Higher power of high-frequent component in the autonomous neural activity in $60\lesssim$ Frequency$\lesssim 80$ [Hz] in 8/1 case in comparison with 4/1.

## Data Availability

Requests to access the datasets should be directed to the corresponding author (nobukawa@cs.it-chiba.ac.jp).

## Appendix

To investigate the characteristics of autonomous spiking activity under the condition of small and large E/I ratios, we evaluated the power spectrum analysis in cases with E/I ratio of 4/1 and 8/1 under absence of external input condition. In the spiking neural network with strong-EPSP, the autonomous spiking activity is maintained even under the absence of external input, known as spontaneous spiking activity (Teramae et al. 2012). Figure 11 shows PSD in cases with an E/I ratio of 4/1 and 8/1 under absence of external input condition. Here, an E/I ratio of 4/1 and 8/1 corresponds to typical low and high E/I ratio cases, respectively. To trigger autonomous neural activity without external input, known as spontaneous activity, the input spike-series produced by a Poisson process ($\varLambda =0.3$ Hz) with an initial duration of $0 \le t\le 100$ ms was applied. From this result, higher power of high-frequent component in the autonomous neural activity in $60\lesssim$ Frequency $\lesssim 80$ [Hz] in 8/1 case in comparison with 4/1. Here, with a smaller E/I ratio less than 4/1, such as 3/1 and 2/1, the spontaneous activity cannot emerge. That is, high E/I ratios enhance the high frequency-component of spontaneous activity involving gamma-frequency components.

## Code availability

The source code for simulating the spiking activity can be found at the following address: https://github.com/SouNobukawa/ASSR_SNN.

## References

[CR1] Bartos M, Vida I, Frotscher M, Meyer A, Monyer H, Geiger JR, Jonas P (2002). Fast synaptic inhibition promotes synchronized gamma oscillations in hippocampal interneuron networks. Proc Natl Acad Sci.

[CR2] Ben-Ari Y (2015). Is birth a critical period in the pathogenesis of autism spectrum disorders?. Nat Rev Neurosci.

[CR3] Berg AT, Plioplys S (2012). Epilepsy and autism: is there a special relationship?. Epilepsy Behav.

[CR4] Börgers C, Kopell N (2003). Synchronization in networks of excitatory and inhibitory neurons with sparse, random connectivity. Neural Comput.

[CR5] Bruns A, Eckhorn R (2004). Task-related coupling from high-to low-frequency signals among visual cortical areas in human subdural recordings. Int J Psychophysiol.

[CR6] Chao HT, Chen H, Samaco RC, Xue M, Chahrour M, Yoo J, Neul JL, Gong S, Lu HC, Heintz N (2010). Dysfunction in gaba signalling mediates autism-like stereotypies and rett syndrome phenotypes. Nature.

[CR7] Chattopadhyaya B, Di Cristo G (2012). Gabaergic circuit dysfunctions in neurodevelopmental disorders. Front Psychiatry.

[CR8] Dehghani N, Peyrache A, Telenczuk B, Le Van Quyen M, Halgren E, Cash SS, Hatsopoulos NG, Destexhe A (2016). Dynamic balance of excitation and inhibition in human and monkey neocortex. Sci Rep.

[CR9] Galambos R, Makeig S, Talmachoff PJ (1981). A 40-Hz auditory potential recorded from the human scalp. Proc Natl Acad Sci U S A.

[CR10] Gao R, Peterson EJ, Voytek B (2017). Inferring synaptic excitation/inhibition balance from field potentials. NeuroImage.

[CR11] Gibson JR, Bartley AF, Hays SA, Huber KM (2008). Imbalance of neocortical excitation and inhibition and altered up states reflect network hyperexcitability in the mouse model of fragile x syndrome. J Neurophysiol.

[CR12] Glausier JR, Lewis DA (2013). Dendritic spine pathology in schizophrenia. Neuroscience.

[CR13] Goodman DF, Stimberg M, Yger P, Brette R (2014). Brian 2: neural simulations on a variety of computational hardware. BMC Neurosci.

[CR14] Gray CM, König P, Engel AK, Singer W (1989). Oscillatory responses in cat visual cortex exhibit inter-columnar synchronization which reflects global stimulus properties. Nature.

[CR15] Grent T, Gross J, Goense J, Wibral M, Gajwani R, Gumley AI, Lawrie SM, Schwannauer M, Schultze-Lutter F, Schröder TN (2018). Resting-state gamma-band power alterations in schizophrenia reveal E/I-balance abnormalities across illness-stages. Elife.

[CR16] Guo D, Wang Q, Perc M (2012). Complex synchronous behavior in interneuronal networks with delayed inhibitory and fast electrical synapses. Phys Rev E.

[CR17] Guo D, Chen M, Perc M, Wu S, Xia C, Zhang Y, Xu P, Xia Y, Yao D (2016). Firing regulation of fast-spiking interneurons by autaptic inhibition. EPL (Europhys Lett).

[CR18] Guo D, Wu S, Chen M, Perc M, Zhang Y, Ma J, Cui Y, Xu P, Xia Y, Yao D (2016). Regulation of irregular neuronal firing by autaptic transmission. Sci Rep.

[CR19] Hájos N, Paulsen O (2009). Network mechanisms of gamma oscillations in the ca3 region of the hippocampus. Neural Netw.

[CR20] Hashemi E, Ariza J, Rogers H, Noctor SC, Martínez-Cerdeño V (2017). The number of parvalbumin-expressing interneurons is decreased in the prefrontal cortex in autism. Cerebral Cortex.

[CR21] Hillyard SA, Mangun GR, Woldorff MG, Luck SJ (1995) Neural systems mediating selective attention

[CR22] Hirano Y, Oribe N, Kanba S, Onitsuka T, Nestor PG, Spencer KM (2015). Spontaneous gamma activity in schizophrenia. JAMA Psychiatry.

[CR23] Hiratani N, Teramae JN, Fukai T (2013). Associative memory model with long-tail-distributed hebbian synaptic connections. Front Comput Neurosci.

[CR24] Hromádka T, DeWeese MR, Zador AM (2008). Sparse representation of sounds in the unanesthetized auditory cortex. PLoS Biol.

[CR25] Izhikevich EM (2003). Simple model of spiking neurons. IEEE Trans Neural Netw.

[CR26] Juergens E, Guettler A, Eckhorn R (1999). Visual stimulation elicits locked and induced gamma oscillations in monkey intracortical-and EEG-potentials, but not in human eeg. Exp Brain Res.

[CR27] Kada H, Teramae JN, Tokuda IT (2018). Highly heterogeneous excitatory connections require less amount of noise to sustain firing activities in cortical networks. Front Comput Neurosci.

[CR28] Kessler K, Seymour RA, Rippon G (2016). Brain oscillations and connectivity in autism spectrum disorders (ASD): new approaches to methodology, measurement and modelling. Neurosci Biobehav Rev.

[CR29] Lefort S, Tomm C, Sarria JCF, Petersen CC (2009). The excitatory neuronal network of the c2 barrel column in mouse primary somatosensory cortex. Neuron.

[CR30] Legget KT, Hild AK, Steinmetz SE, Simon ST, Rojas DC (2017). MEG and EEG demonstrate similar test-retest reliability of the 40 HZ auditory steady-state response. Int J Psychophysiol.

[CR31] McNally JM, McCarley RW (2016). Gamma band oscillations: a key to understanding schizophrenia symptoms and neural circuit abnormalities. Curr Opin Psychiatry.

[CR32] Naatanen R, Näätänen R (1992). Attention and brain function.

[CR33] Nobukawa S, Nishimura H, Yamanishi T (2019). Temporal-specific complexity of spiking patterns in spontaneous activity induced by a dual complex network structure. Sci Rep.

[CR34] Nobukawa S, Nishimura H, Wagatsuma N, Ando S, Yamanishi T (2020) Long-tailed characteristic of spiking pattern alternation induced by log-normal excitatory synaptic distribution. IEEE Trans Neural Netw Learn Syst 10.1109/TNNLS.2020.3015208 32822305

[CR35] Oda Y, Onitsuka T, Tsuchimoto R, Hirano S, Oribe N, Ueno T, Hirano Y, Nakamura I, Miura T, Kanba S (2012). Gamma band neural synchronization deficits for auditory steady state responses in bipolar disorder patients. PLoS ONE.

[CR36] O’Donnell BF, Hetrick WP, Vohs JL, Krishnan GP, Carroll CA, Shekhar A (2004). Neural synchronization deficits to auditory stimulation in bipolar disorder. Neuroreport.

[CR37] O’Donnell BF, Vohs JL, Krishnan GP, Rass O, Hetrick WP, Morzorati SL (2013) The auditory steady-state response (assr): a translational biomarker for schizophrenia. In: Supplements to Clinical neurophysiology, vol 62, Elsevier, pp 101–112 10.1016/b978-0-7020-5307-8.00006-5 PMC4959266 24053034

[CR38] Omura Y, Carvalho MM, Inokuchi K, Fukai T (2015). A lognormal recurrent network model for burst generation during hippocampal sharp waves. J Neurosci.

[CR39] Penttonen M, Kamondi A, Acsády L, Buzsáki G (1998). Gamma frequency oscillation in the hippocampus of the rat: intracellular analysis in vivo. Eur J Neurosci.

[CR40] Powell EM, Campbell DB, Stanwood GD, Davis C, Noebels JL, Levitt P (2003). Genetic disruption of cortical interneuron development causes region-and GABA cell type-specific deficits, epilepsy, and behavioral dysfunction. J Neurosci.

[CR41] Rass O, Krishnan G, Brenner CA, Hetrick WP, Merrill CC, Shekhar A, O’Donnell BF (2010). Auditory steady state response in bipolar disorder: relation to clinical state, cognitive performance, medication status, and substance disorders. Bipolar Dis.

[CR42] Rojas DC, Teale PD, Maharajh K, Kronberg E, Youngpeter K, Wilson LB, Wallace A, Hepburn S (2011). Transient and steady-state auditory gamma-band responses in first-degree relatives of people with autism spectrum disorder. Mol Autism.

[CR43] Rubin R, Abbott L, Sompolinsky H (2017). Balanced excitation and inhibition are required for high-capacity, noise-robust neuronal selectivity. Proc Natl Acad Sci.

[CR44] Sakata S, Harris KD (2009). Laminar structure of spontaneous and sensory-evoked population activity in auditory cortex. Neuron.

[CR45] Seymour RA, Rippon G, Gooding-Williams G, Sowman PF, Kessler K (2020). Reduced auditory steady state responses in autism spectrum disorder. Mol Autism.

[CR46] Softky WR, Koch C (1993). The highly irregular firing of cortical cells is inconsistent with temporal integration of random EPSPs. J Neurosci.

[CR47] Song S, Sjöström PJ, Reigl M, Nelson S, Chklovskii DB (2005). Highly nonrandom features of synaptic connectivity in local cortical circuits. PLoS Biol.

[CR48] Spence SJ, Schneider MT (2009). The role of epilepsy and epileptiform EEGs in autism spectrum disorders. Pediatric Res.

[CR49] Spencer KM, Salisbury DF, Shenton ME, McCarley RW (2008). $$\gamma$$-band auditory steady-state responses are impaired in first episode psychosis. Biol Psychiatry.

[CR50] Takahashi T, Yamanishi T, Nobukawa S, Kasakawa S, Yoshimura Y, Hiraishi H, Hasegawa C, Ikeda T, Hirosawa T, Munesue T (2017). Band-specific atypical functional connectivity pattern in childhood autism spectrum disorder. Clin Neurophysiol.

[CR51] Takahashi T, Goto T, Nobukawa S, Tanaka Y, Kikuchi M, Higashima M, Wada Y (2018). Abnormal functional connectivity of high-frequency rhythms in drug-naïve schizophrenia. Clin Neurophysiol.

[CR52] Tallon-Baudry C, Bertrand O, Delpuech C, Pernier J (1996). Stimulus specificity of phase-locked and non-phase-locked 40 HZ visual responses in human. J Neurosci.

[CR53] Tan HR, Gross J, Uhlhaas P (2015). Meg-measured auditory steady-state oscillations show high test-retest reliability: a sensor and source-space analysis. NeuroImage.

[CR54] Teramae JN, Tsubo Y, Fukai T (2012). Optimal spike-based communication in excitable networks with strong-sparse and weak-dense links. Sci Rep.

[CR55] Uhlhaas PJ, Singer W (2012). Neuronal dynamics and neuropsychiatric disorders: toward a translational paradigm for dysfunctional large-scale networks. Neuron.

[CR56] Uhlhaas PJ, Haenschel C, Nikolić D, Singer W (2008). The role of oscillations and synchrony in cortical networks and their putative relevance for the pathophysiology of schizophrenia. Schizophrenia Bull.

[CR57] Watanabe K, Teramae JN, Wakamiya N (2016) Inferred duality of synaptic connectivity in local cortical circuit with receptive field correlation. In: International conference on neural information processing, Springer, pp 115–122

[CR58] Whittington MA, Traub R, Kopell N, Ermentrout B, Buhl E (2000). Inhibition-based rhythms: experimental and mathematical observations on network dynamics. Int J Psychophysiol.

[CR59] Wilson TW, Rojas DC, Reite ML, Teale PD, Rogers SJ (2007). Children and adolescents with autism exhibit reduced MEG steady-state gamma responses. Biol Psychiatry.

[CR60] Zhou TH, Mueller NE, Spencer KM, Mallya SG, Lewandowski KE, Norris LA, Levy DL, Cohen BM, Öngür D, Hall MH (2018). Auditory steady state response deficits are associated with symptom severity and poor functioning in patients with psychotic disorder. Schizophrenia Res.

